# Electroacupuncture Attenuates Visceral Hypersensitivity by Inhibiting JAK2/STAT3 Signaling Pathway in the Descending Pain Modulation System

**DOI:** 10.3389/fnins.2017.00644

**Published:** 2017-11-20

**Authors:** Juan Wan, Yi Ding, Adnan H. Tahir, Manoj K. Shah, Habibullah Janyaro, Xiaojing Li, Juming Zhong, Vitaly Vodyanoy, Mingxing Ding

**Affiliations:** ^1^Department of Clinical Veterinary Medicine, College of Veterinary Medicine, Huazhong Agricultural University, Wuhan, China; ^2^Department of Anatomy, Physiology and Pharmacology, Auburn University, Auburn, AL, United States

**Keywords:** electroacupuncture (EA), TNBS, visceral hypersensitivity (VH), visceromotor response (VMR), JAK2/STAT3 signaling pathway, PAG-RVM-SCDH axis

## Abstract

Electroacupuncture (EA) has been used for treating visceral hypersensitivity (VH). However, the underlying molecular mechanism remains unclear. This study was aim to testify the effect of EA on ileitis-provoked VH, and to confirm whether EA attenuates VH through Janus kinase 2 (JAK2)/signal transducers and activators of transcription 3 (STAT3) signaling pathway in the periaqueductal gray (PAG)-the rostral ventromedial medulla (RVM)-the spinal cord dorsal horn (SCDH) axis.

**Methods:** Goats were anesthetized and laparotomized for injecting 2,4,6-trinitro-benzene-sulfonic acid (TNBS)-ethanol solution (30mg TNBS dissolved in 40% ethanol) into the ileal wall to induce VH. EA was treated for 30min from day 7, then every 3 days for six times. VH was assessed by visceromotor response (VMR) and pain behavior response to 20, 40, 60, 80, and 100 mmHg colorectal distension pressures at day 7, 10, 13, 16, 19, and 22. The spinal cord in the eleventh thoracic vertebra and the brain were collected at day 22. The protein and mRNA levels of IL-6, JAK2, and STAT3 in the SCDH were detected with western blot and qPCR, respectively. The distribution of these substances was observed with immunohistochemistry in the ventrolateral PAG (vlPAG), RVM (mainly the nucleus raphe magnus, NRM), SCDH, the nucleus tractus solitaries (NTS) and the dorsal motor nucleus of vagi (DMV).

**Results:** Goats administered with TNBS-ethanol solution showed diarrhea, enhanced VMR and pain behavior response, and increased IL-6, phosphorylated JAK2 and STAT3 (pJAK2 and pSTAT3) in the vlPAG, NRM, NTS and DMV, and their protein and mRNA levels in the SCDH. EA relieved diarrhea, VMR and pain behavior response, decreased IL-6, pJAK2 and pSTAT3 levels in the vlPAG, NRM, SCDH, NTS, and DMV except for pSTAT3 in the DMV, but did not affect mRNA level of these three substances in the SCDH.

**Conclusion:** EA attenuates VH probably through inhibiting JAK2/STAT3 signaling pathway in the PAG-RVM-SCDH axis.

## Introduction

Inflammatory bowel diseases (IBDs) are a collection of chronic inflammatory disorders of the gastrointestinal tract in human and animals. They lead to discomforts, such as diarrhea, nausea, abdominal pain, and sometimes visceral hypersensitivity (VH) (Rao et al., 1987; Loening-Baucke et al., 1989; Bernstein et al., 1996; Bross et al., 1996; Atreya et al., 2000; Chang et al., 2000; Faure and Giguere, 2008; Van Hoboken et al., 2011; Rubio et al., 2014). VH is associated with altered bowel movement, increased mucosal secretion and a lowered sensory threshold to colorectal distention in human and animals (Drossman, 1999). It has been supposed that VH is caused by triggering events, such as injury, inflammation, with the release of ensuing various neurogenic factors, provoking sustained depolarization of the primary afferent nerve terminals, and hereby exciting the spinal and supraspinal neurons (central sensitization) (Price et al., 2006; Latremoliere and Woolf, 2009). There are about 50–70% of people worldwide suffering VH during the IBD development or for the whole lifelong after IBD fades away (Longstreth et al., 2003; Spiegel, 2009). VH seriously impairs their life quality. In order to find an effective treatment to VH, it is essential to understand the central molecular regulating mechanisms underlying VH.

Janus kinase (JAK)/signal transducers and activators of transcription (STAT) pathway which is activated by cytokines is the key signaling pathway in central sensitization (Nicolas et al., 2012) and attracts more attention in recent years. JAKs comprise four tyrosine kinases (JAK1, JAK2, JAK3 and TYK2) while STATs contain seven transcription factors (STAT1, STAT2, STAT3, STAT4, STAT5A, STAT5B, and STAT6). Among JAKs, JAK2 is expressed at a high level in the central nerve system (CNS) (De-Fraja et al., 1998), and can specifically activate STAT3 (Sriram et al., 2004). Bradesi et al. (2015) found that the activation of STAT3 in the spinal cord was involved in the development of VH. Tsuda et al. (2011) reported that nerve injury activated JAK/STAT3 pathway and induced hypersensitivity, and intrathecal administration of JAK/STAT3 signal inhibitors reversed the hypersensitivity. Interleukin 6 (IL-6) is one of the important upstream proinflammatory cytokines of JAK2/STAT3 pathway. DeLeo et al. (1996) suggested that IL-6 was involved in hypersensitivity that arises from neuropathic pain. Arruda et al. (2000) and Dominguez et al. (2008) verified that intrathecal injection of anti-IL-6 antibody prevented the spinal nerve lesion-induced hypersensitivity and accumulation of phosphorylated STAT3 (pSTAT3) in the spinal cord. These findings indicate that JAK2/STAT3 signaling pathway activated by IL-6 facilitates the development of hypersensitivity. Therefore, JAK2/STAT3 signaling pathway may be potential therapeutic targets for treatment of hypersensitivity in human and animals.

There are several drugs for VH treatment in clinic. Nonsteroid anti-inflammatory drugs and opioid peptides are most used. Because of their poor analgesia effect or drug dependence, new therapies are still in urgent need. Acupuncture has shown to be effective for various painful disorders in humans or animals (Cui et al., 2016; Liu et al., 2017; Wu et al., 2017; Zhao et al., 2017), including chronic pain (Chan et al., 1997; Xiao and Liu, 2004; Zeng et al., 2016). Electroacupuncture (EA, a modern version of acupuncture) has been demonstrated to reduce the amplitude of electromyograms from the rectus abdominis in response to colorectal distention stimulation in rats (Cui et al., 2005) and mice (Wang et al., 2012), may become a promising supplementary treatment for VH. Studies showed that EA induced analgesia through activating (or inhibiting) the descending inhibitory (or facilitatory) system, mainly including periaqueductal gray (PAG)-rostral ventromedial medulla (RVM)-the spinal cord dorsal horn (SCDH) (Zhi-Qi, 2008). So far the specific mechanism by which EA relieves VH is not clear. Liu et al. (2012) used EA to treat cerebral ischemia and found that EA can reduce the expression of phosphorylated JAK2 (pJAK2) and pSTAT3 in the CNS. However, whether EA modifies VH through JAK2/STAT3 signaling pathway in the PAG-RVM-SCDH axis is not reported.

Electroacupuncture (EA) induced-analgesia (EAA) in goats (ruminants) is superior to that in rats or human (Han, 1997; Liu et al., 2009a; Shah et al., 2016b). Goats are optimal animals for studying the mechanism underlying EAA. In the present study, VH was induced by ileitis, through 2,4,6-trinitro-benzene-sulfonic acid (TNBS)/ethanol solution injected into the ileum wall of goats. EA was used to stimulate bilateral Housanli points of the goats with VH every 3 days for total 6 times. Pain behavior response and visceral motor response (VMR) were provoked with colorectal distension (CRD). The expression levels of IL-6, pJAK2 and pSTAT3 in the CNS were detected. This experiment was to explore the central regulating mechanism by which EA controls VH.

## Materials and methods

### Animals and groups

The study was conducted under the guidelines approved by Institutional Animal Care and Use Committee of the Huazhong Agricultural University, Wuhan, China and adhered to the guidelines of the Committee for Research and Ethical Issues of the International Association for the Study of Pain. (Permit number: HZAUGO-2016-005).

Thirty 1-year-old hybrid male goats, weighing 20 ± 2 kg, were randomly allocated into 4 treatment groups: Saline (*n* = 9), TNBS (*n* = 9), TNBS + sham EA (Sham, *n* = 6), and TNBS + EA (EA, *n* = 6) groups. All goats were purchased from the Hubei Agricultural Academy of Science Animals, were fed by a dry grass diet supplemented with a cereal-based concentrate, and drank freely. They were dewormed and acclimatized to the surrounding for 2 weeks before the initiation of the study. The experiment was performed in a quiet environment, with a temperature of 20–25°C.

### Visceral hypersensitivity model

**Figure 1:**
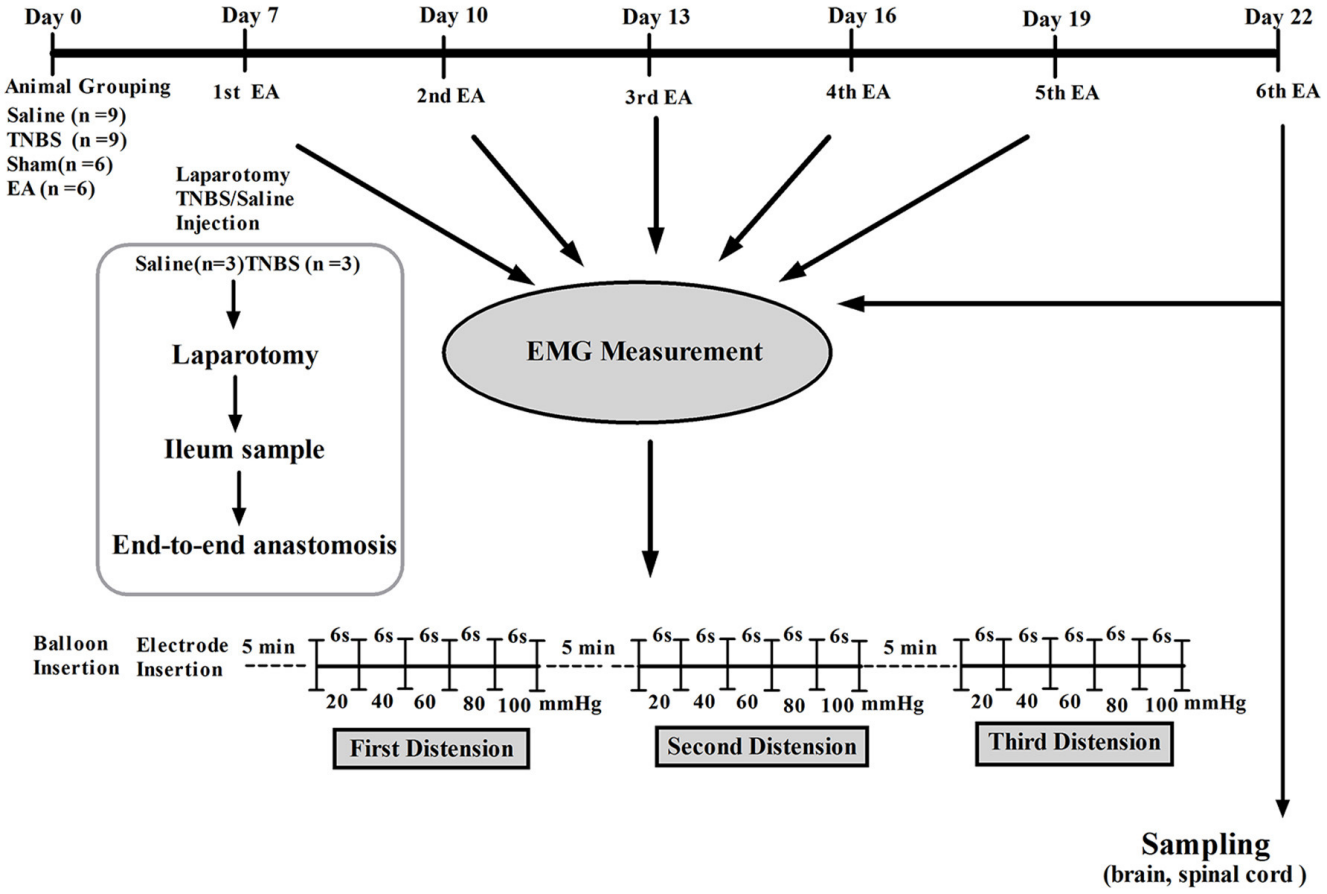
The scheme of experiment.

Visceral hypersensitivity (VH) was established with the method described by Hassan et al. (2015). Briefly, the goats were kept off feed to avoid regurgitation and respiratory complications for 24 h prior to the experiment. Baseline cardinal parameters like respiratory rates, pulse rates and body temperature were recorded. The goats were intramuscularly (IM) injected with atropine (0.03 mg/kg, Tianjin Pharmaceutical Group Xinzheng Co., Ltd, China), etamsylate (0.02 g/kg, Shandong Fangming Pharmaceutical Co., Ltd, China) and xylazole (0.1 mg/kg, Hebei Gaocheng Sihai Pharmaceutical Industry Co., Ltd, China) before the anesthesia. Goats were placed on left lateral recumbency. Anesthesia was induced by intravenous (IV) administration of ketamine HCl (0.5 mg/kg/min; Yao Pharma Co., Ltd, China). The anesthetic depth was judged according to eyelid reflex, obtunded corneal reflexes and painful response to the prickle of the abdomen skin and coronary hooves. The trachea was intubated to avoid aspiration of ruminal contents. Lidocaine 2% (Shandong Hualu Pharmaceutical Co., Ltd, China) was used by local infiltration anesthesia. During the surgery, IV infusion (rate 0.05 mg/kg/min) of ketamine was conducted when the goats moved or struggled and until they entered a shallow anesthetic state. Following aseptic surgical procedure, a 6-cm abdominal incision was opened in the right flank to locate the ileum, and its terminal part was exteriorized gently on moist sterile gauze. For TNBS group, 1.2 ml of TNBS (Sigma Aldrich, USA) solution (30 mg TNBS dissolved in 40% ethanol) was injected into the wall of the ileum via five points with a 30 gauge needle, approximately 15 cm proximal to the ileocecal junction. In the case of saline group, the same volume of normal saline (0.9% NaCl) was injected in the same manner as in the TNBS group. The intestine was then returned to the abdominal cavity, the abdominal wall and peritoneum were closed with cat gut sutures, and the skin was closed with silk sutures. The goats were housed individually and monitored during the recovery from anesthesia (1–2 h). To prevent the pain, the goats were given tramadol (5 mg/kg, IM, Hubei Qianjiang Pharmaceutical Co., Ltd, China). The post-operative condition of the goats was monitored at least twice daily. The wound was treated with iodophor (Wuhan Xuehuan Sterilization Goods Co., Ltd, China) and erythromycin ointment (North China pharmaceutical Co., Ltd, China) daily till completely healed. The scheme of experiment is illustrated in Figure 1.

### Assessment of ileal inflammation

Symptoms such as water/food consumption, mortality, stool consistency, and bloody feces were observed during the experiment. Body weight was recorded at day 0, 7, and 22. Three goats from saline group and another three from TNBS group were taken for a similar laparotomy on day 7. Six-Centimeter terminal ileum of each goat was excised 15 cm proximal to the ileocecal junction, flushed with phosphate buffered saline for detection the macroscopic changes. Macroscopic lesions were scored by two independent observers who were unaware of the treatments according to a scale of 0–10 scores described by Hassan et al. (2015). After that, a 2 × 2 cm tissue block was taken from the approximate part of the ileum segment opposite to the mesentery, and fixed in 4% buffer formaldehyde for hematoxylin-eosin (HE) staining. Another ileal tissue (2 × 2 cm) was weighed, frozen in liquid nitrogen and finally transferred to store in −80°C for measuring the myeloperoxidase (MPO) concentration. Immediately, an end-to-end anastomosis was conducted and the abdomen was closed using standard methods. These six goats received tramadol hydrochloride (5 mg/kg, IM) and ampicillin (20 mg/kg, IM) for 5 days. After two-week recovery, the animals were sent to a goat farm.

Ileal tissues fixed in 4% buffer formaldehyde were embedded in paraffin. The tissue block was sectioned at a thickness of 5 μm, and mounted on poly-lysine coated slides. Three serial slides were deparaffined, rehydrated sequentially and stained with hematoxylin and eosin. The slides were visualized with the images under a light microscope (Nikon ECLIPSE 80I, Nikon Corporation, and Tokyo, Japan) with a 10 × and 40 × objective lens. Microscopic changes were blindly assessed by two investigators using a scoring system 0–10 as previously described (Hassan et al., 2015). Another ileal tissue (2 × 2 cm) was grinded and homogenized in 1 ml PBS, pH 7.2 at 4°C. The solution was centrifuged at 5,000 g at 4°C for 10 min. The protein concentration of the supernatant was determined using Nanodrop Spectrophotometer (Thermo Fisher Scientific, Inc., USA). The MPO concentration was assayed with MPO ELISA Kit (eBioscience, Inc., San Diego, CA 92121, USA) according to the manufacturer's instruments. Each sample was analyzed in triplicate and the values were presented as pg/mg.

### EA treatment

Electroacupuncture (EA) treatment started on day 7, then every 3 days for total 6 times according to the method described by Wang et al. (2002). For EA treatment, the set of bilateral Housanli acupoints (equal to Zusanli in humans) was selected for EA groups. Here the acupoints are nominated with Pinyin Naming System instead of the Meridian Numbering System because animal's meridians are not completely recorded. The anatomic location and use of these points have been described in detail in veterinary medicine (Qiu et al., 2015; Hu et al., 2016). Prior to the EA treatment, goats were restrained. The acupoint sites were shaved and disinfected with 75% ethanol. Needles were disinfected and inserted into the points by a skilled acupuncturist. A pair of stainless steel needles was perpendicularly (0.40 mm in diameter and 50 mm in length) inserted at a depth of 20–30 mm into bilaterally Housanli acupoints (in the muscular groove between the long digital extensor and the lateral digital extensor muscles below the head of the fibula on the lateral surface) of each hind leg. The needles in set of EA group were connected by a pair of wires to one output of WQ-6F Electronic Acupunctoscope (Beijing Xindonghua Electronic Instrument Co., Ltd., China). Stimulation with constant wave of 60 Hz (approximate 3.2 V) was applied and lasted for 30 min each time according to Cheng et al.'s method (Cheng et al., 2012). The goats in the other three groups were restrained in the same manner as the EA-treated goats. Sham goats underwent needle placement without electric stimulation.

### Colorectal distension testing

Visceral hypersensitivity (VH) was reflected by EMG and pain response score. EMG was performed to record VMR to CRD of goats immediately after termination of EA on day 7, 10, 13, 16, 19, and 22 after induction of ileitis as described previously (Hassan et al., 2015). Briefly, prior to EMG record, the goats were standing, two nickel-steel needles (0.45 mm in diameter and 5 cm in length) were inserted 2 cm apart into external oblique muscles at the center of the left abdomen as electrodes, both electrodes were clamped and connected to an EMG recorder (Nanjing Medease Science and Technology Co., Ltd, China). EMG was amplified and filtered with a processing system MedLab-U/4C501H (Nanjing Medease Science and Technology Co., Ltd, China). A manual distension device was made with a T-connector connecting a balloon, a vacuum pump and a sphygmomanometer (**Figure 6H**). The polyethylene balloon (12 cm) was lubricated and inserted into the distal colon 10 cm from the anus of goats. After goats were acclimatized for 10 min, the balloon was inflated with the pump. The pressure in the balloon was measured with the sphygmomanometer. It continuously increased from 20 to 40, 60, 80, and 100 mmHg by stage and lasted for 6 s at each stage. EMG was recorded during each stage. This procedure was repeated every 5 min for 3 times. The area under curve (AUC) was calculated from EMG data with MedLabV6.3 software (Nanjing medease science and technology Co., Ltd, China) and expressed as millivolt multiply second (mv^*^s).

Pain response scores were assessed through monitoring pain behavioral responses to CRD. The modified pain behavioral scale of 0–4 was used for this study (Habibullah et al., 2016). The behavior response to CRD distension was observed by two observers blinded to the experimental conditions. The pain behavior responses to different CRD distensions were assessed three times and averaged as pain scores.

### Sample collection

**Figure 2:**
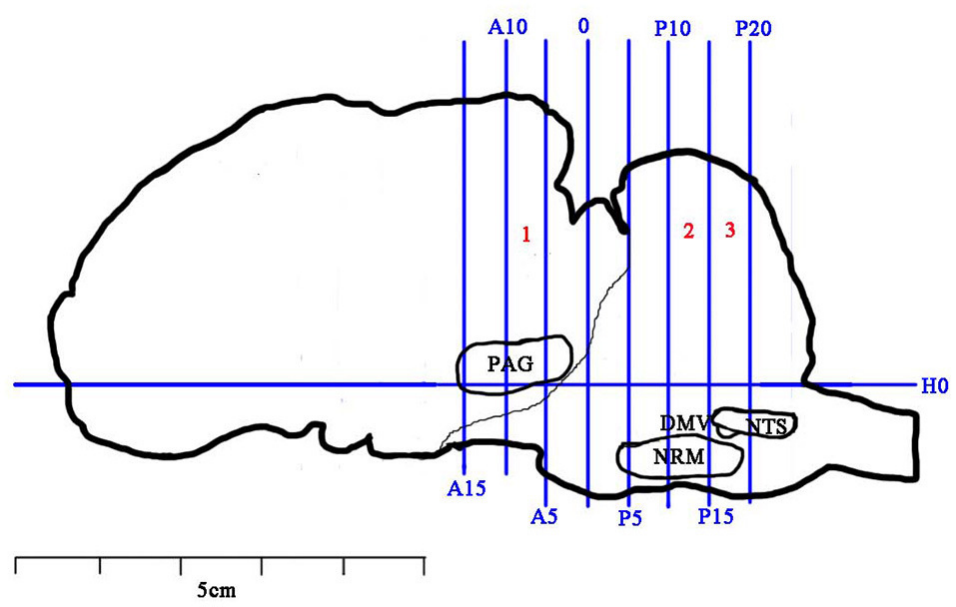
Brain sectioning. H0: the horizontal zero plane. A15, A10, A5, and 0 show transverse planes at 15, 10, 5, and 0 mm anterior to the interaural line, and P5, P10, P15, and P20 show transverse planes at 5, 10, 15, and 20 mm posterior to the interaural line, respectively. The nuclei and areas identifed include the nuclei or areas to be observed were the periaqueductal gray (PAG), the nucleus raphe magnus (NRM), the nucleus tractus solitarius (NTS) and the dorsal motor nucleus of vagi (DMV).

Immediately after the last CRD, the goats were anesthetized with intravenously administered xylidinothiazoline at 3 mg/kg. The spinal cord of the eleventh thoracic vertibrae (T11) was removed very carefully and divided into two parts. The anterior part was obtained and stored in 10% formalin for immunohistochemistry (IHC) and the posterior part was weighed, frozen in liquid nitrogen and finally transferred to store in −80°C for western blotting and RT-PCR analysis. Then, the heads of the goats were taken and mounted onto a stereotaxic instrument (Tindal et al., 1968, 1987). The brain was taken out for observing IL-6, pJAK2 and pSTAT3 in the descending pain system (including ventrolateral PAG (vlPAG), RVM (mainly the nucleus raphe magnus, NRM) and SCDH), and nuclei related to visceral motion and sensory regulations (including the nucleus tractus solitaries (NTS) and the dorsal motor nucleus of vagi (DMV)) with IHC. RT-PCR and western blotting methods were not applied for brain tissues because these substances are not evenly distributed in the specific pain-regulated areas and their inaccurate sampling would result in homogenization. The brain was divided into 3 blocks (B1-B3) according to Qiu et al.'s (2015) and Hu et al.'s (2016) methods. The vertical interaural plane represented the zero reference point for the anterior-posterior coordinates, and the horizontal zero plane (H0) intersected the interaural point and a point 25 mm above the lower margin of the orbit. B1-B3 are from anterior 10 mm (A10) to anterior 5 mm (A5), posterior 10 mm (P10) to posterior 15 mm (P15), and posterior 15 mm (P15) to posterior 20 mm (P20), respectively (Figure 2). The locations of the nuclei or areas and the morphological characteristics of the neurons were identified according to the brain atlas of goats and pigs (Dean et al., 1999; Félix et al., 1999).

### Immunohistochemistry

**Figure 3:**
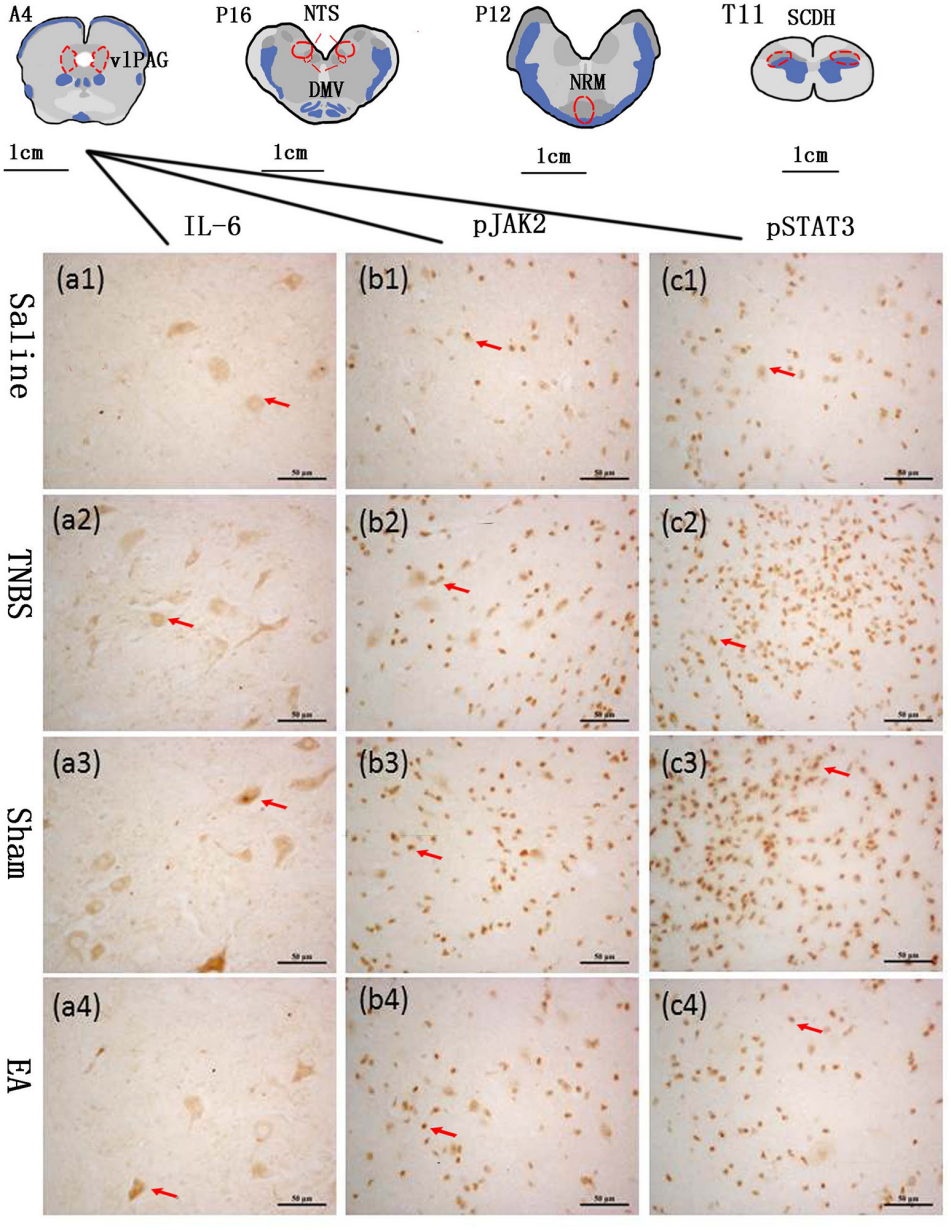
The nuclei (areas) locations used for the IL-6-like, pJAK2-like and pSTAT3-like immunoreactive (IL-6-IR, pJAK2-IR and pSTAT3-IR) cells counts and the representative cells in the vlPAG. A6: the ventrolateral periaqueductal gray (vlPAG) at interaural levels of 6 mm. P12: the nucleus raphe magnus (NRM) at interaural levels of −12 mm. P16: the nucleus tractus solitarius (NTS) and the dorsal motor nucleus of vagi (DMV) at interaural levels of −16 mm. T11: the anterior part of the eleventh thoracic vertebra spinal cord dorsal horn (SCDH). (a1–a4) show positive IL-6-IR cells in the vlPAG in Saline, TNBS, Sham and EA group, respectively. (b1–b4) show positive pJAK2-IR cells in the vlPAG in Saline, TNBS, Sham and EA group, respectively. (c1–c4) show positive pSTAT3-IR cells in the vlPAG in Saline, TNBS, Sham and EA group, respectively. Arrows point to the positive cells. The cytoplasm or nuclei of positive cells was stained as brown yellow. The bars = 50 μm.

The blocks and T11 spinal cord were embedded in paraffin with their rostral surface facing up. Each of the blocks was consecutively sectioned at a thickness of 5 μm. Ten serial slides of each nucleus or area were mounted on polylysine coated slides, deparafnized, and rehydrated sequentially. Three of these 10 slides were incubated with rabbit-anti-IL-6 IgG (CST, Inc., USA, #12153, diluted 1:100 in PBS), rabbit anti-pJAK2 IgG (CST, Inc., USA, #3776, diluted 1:200 in PBS) or rabbit anti-pSTAT3 IgG (CST, Inc., USA, #9145, diluted 1:200 in PBS), respectively. One slide was incubated with PBS instead of the corresponding antibody as negative control. Experimental procedures of SABC immunohistochemistry followed the instructions provided by the reagent company (Boster biotech, Wuhan, China). The cytoplasm or nuclei of positive cells was stained as brown yellow. The locations of the observed nuclei (areas) with the representative stained cells are shown in Figure 3.

Optical images of the stained nuclei or area in the CNS were obtained under a light microscope (Nikon ECLIPSE 80I, Nikon Corporation, and Tokyo, Japan) connected to a video-based and computer-linked system (high-resolution pathological image analysis system 1000, Wuhan Qianping Ltd., Wuhan, China). Three fields of each nucleus or brain area on each side were observed with a 40× objective lens. The optical density values of the IL-6-like, pJAK2-like and pSTAT3-like immunoreactive cells were calculated with the Image-Pro plus 6.0 system (Media Cybernetics, Inc., Bethesda, MD, USA).

### Western blotting

The posterior segment of T11 spinal dorsal horn was weighed, grinded in liquid nitrogen, then protein extracted from the grounded spinal cord using the RIPA buffer according to the manufacturer's instruction (Beyotime Biotech, Nantong, China). The protein concentration was measured by Nano Drop Spectrophotometer (Thermo Fisher Scientific, Inc., USA). Equal amounts of protein (40 μg) sample was loaded in 15% SDS polyacrylamide gel and transferred to a PVDF membrane using the Mini-PROTEIN Tetra Cell (Bio-Rad, Hercules, CA, USA). The membrane was blocked for 2 h at the room temperature in 5% BSA and was subsequently immunolabeled overnight at 4°C with primary antibodies, rabbit anti-IL-6 IgG (1:500), rabbit anti-pJAK2 IgG (1:500), rabbit anti-pSTAT3 IgG (1:500) (CST, Inc., USA) or rabbit anti-beta-actin (1:300, Santa Cruz, CA, USA), respectively. The membrane was washed and treated with horseradish-peroxidase-conjugated anti-rabbit secondary antibody (1:5,000, Boster biotech, Wuhan, China) for 1 h at the room temperature. Visualization of the antigen-antibody complex was conducted with a horseradish peroxidase substrate (Millipore, Billerica, MA, USA) using the ImageQuant LAS 4,000 min CCD camera (GE Healthcare, Piscataway Township, NJ, USA). The bands were analyzed by Quantity One software (Bio-Rad). Beta-actin was used as the internal control. Values of these substances were represented as the ratio of the optical density of the bands to the density of the related beta-actin band.

### RT-PCR

Total RNA was extracted from dorsal spinal cord of each group by using Trizol reagent (Invitrogen, Carlsbad, CA, USA). Subsequently, cDNA was synthesized from 900 ng of total RNA using a First Strand cDNA Synthesis Kit (TOYOBO, Osaka, Japan). The primer sequences of IL-6, JAK2, STAT3 and GAPDH are shown in Table 1. RT-PCR was performed with Step One Plus™ Real-Time PCR System (Applied Biosystems, CA, USA) using SYBER Green RT-PCR kit (Takara Dalian, China). The mRNA of IL-6, JAK2 and STAT3 relative to GAPDH mRNA were quantified with the 2^−ΔCt^ method, where ΔCt = Ct _target gene_ - Ct _GAPDH_.

**Table 1:** Primer sequences of IL-6, JAK2, STAT3 and GAPDH.

**Name**	**Accession number**	**Primer sequence**
IL-6	NM_173923.2	F:5′-TTCAGTCCACTCGCTGTCTC-3′
		R:5′-TGCTTGGGGTGGTGTCATTC-3′
JAK2	XM_015464499.1	F:5′-CCAAAGGAGGAGGCATTCGG-3′
		R:5′-CAAACTGCATCAATGAATGGTGTC-3′
STAT3	NM_001012671.2	F:5′-GGGATCTGGCGACAAGGAAT-3′
		R:5′-ATGGGACTTTCACCAGGCTC-3′
GAPDH	XM_005680968.3	F:5′-TGTTTGTGATGGGCGTGAACCA-3'
		R:5′-ATGGCGTGGACAGTGGTCATAA-3'

### Statistical analysis

All data were expressed as mean ± SD. Statistical analyses were performed using SPSS version 18.0 (SPSS Inc., Chicago, IL, USA). The statistical comparisons for parametric data (body weight changes, MPO, VMR, IHC, RT-PCR and western blot) were carried out using one-way analysis of variance (ANOVA) followed by Bonferroni post hoc test. The statistical differences of non-parametric values (macroscopic scores, microscopic scores and pain behavior response) among groups were identified using Kruskal-Wallis ANOVA followed by the rank-based Mann-Whitney U-test. A difference was considered significant if P was less than 0.05.

## Results

### Ileal inflammation

**Figure 4:**
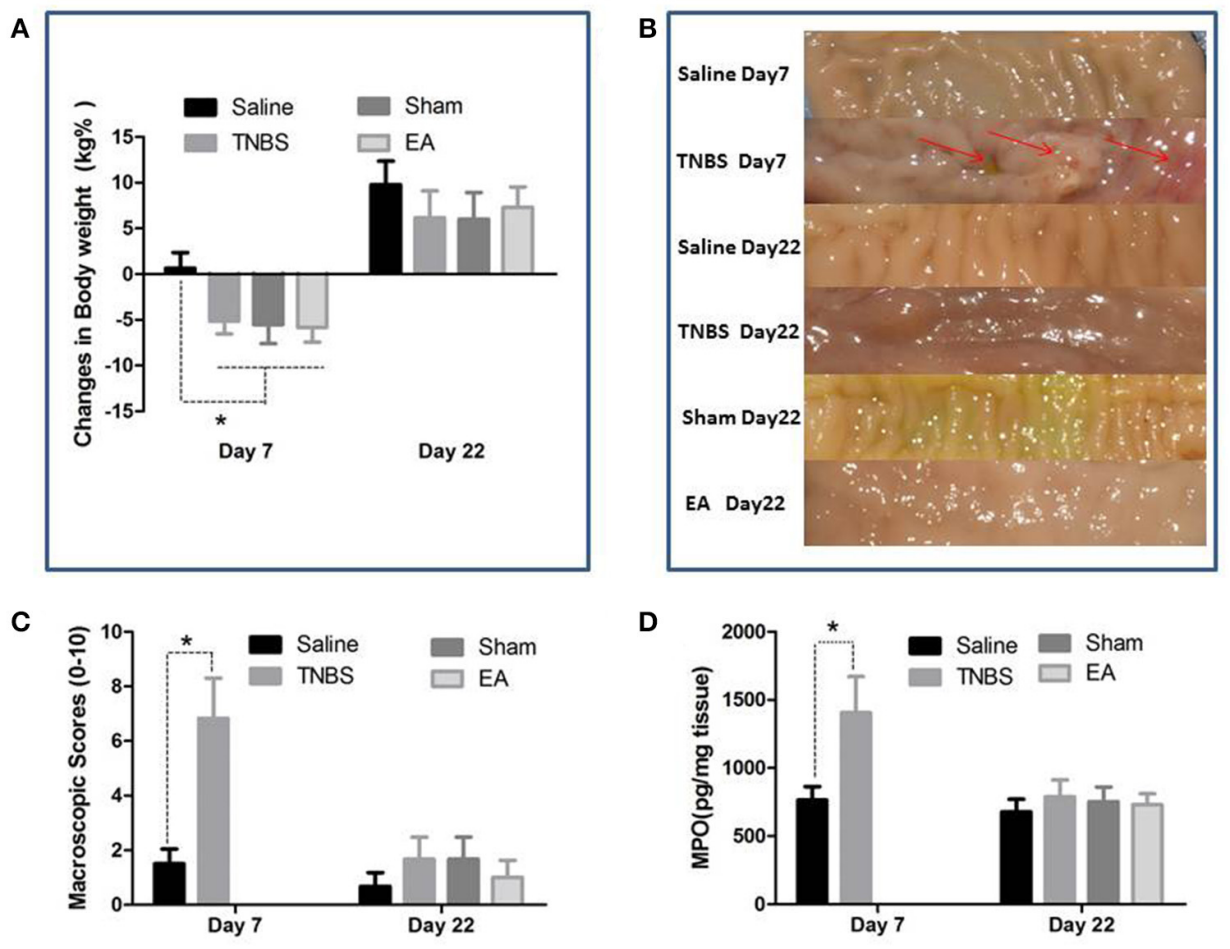
Effects of ileitis and EA treatments on body weight, macroscopic lesions and MPO concentration. **(A)** Body weight changes at day 7 and 22 (mean ± SD, *n* = 6). ^*^P < 0.05 One-way ANOVA followed by Bonferroni's post-test. **(B)** Macroscopic pathologic changes at day 7 and 22. Arrows point to the adhesion, mucosal hyperemia or ulcer. **(C)** Macroscopic change scores on a scale of 0–10 at day 7 (mean ± SD, *n* = 3) and 22 (mean ± SD, *n* = 6). adhesions (0–2), mucosal hyperemia (0–3), ulcers (0–3), wall thickness (0–2). ^*^P < 0.05 Kruskal-Wallis analysis followed by Mann-Whitney U-test. **(D)** MPO concentrations in ileal tissue at day 7 (mean ± SD, *n* = 3) and 22 (mean ± SD, *n* = 6). ^*^P < 0.05 One-way ANOVA followed by Bonferroni's post-test.

The TNBS-treated goats showed inappetence, sluggishness, mucoid diarrhea and weight loss at day 7. The body weight of goats in TNBS, sham and EA groups was decreased (*P* < 0.05) as compared to goats in saline group at day 7. No difference in body weight was found among all groups at day 22 (Figure 4A).

**Figure 5:**
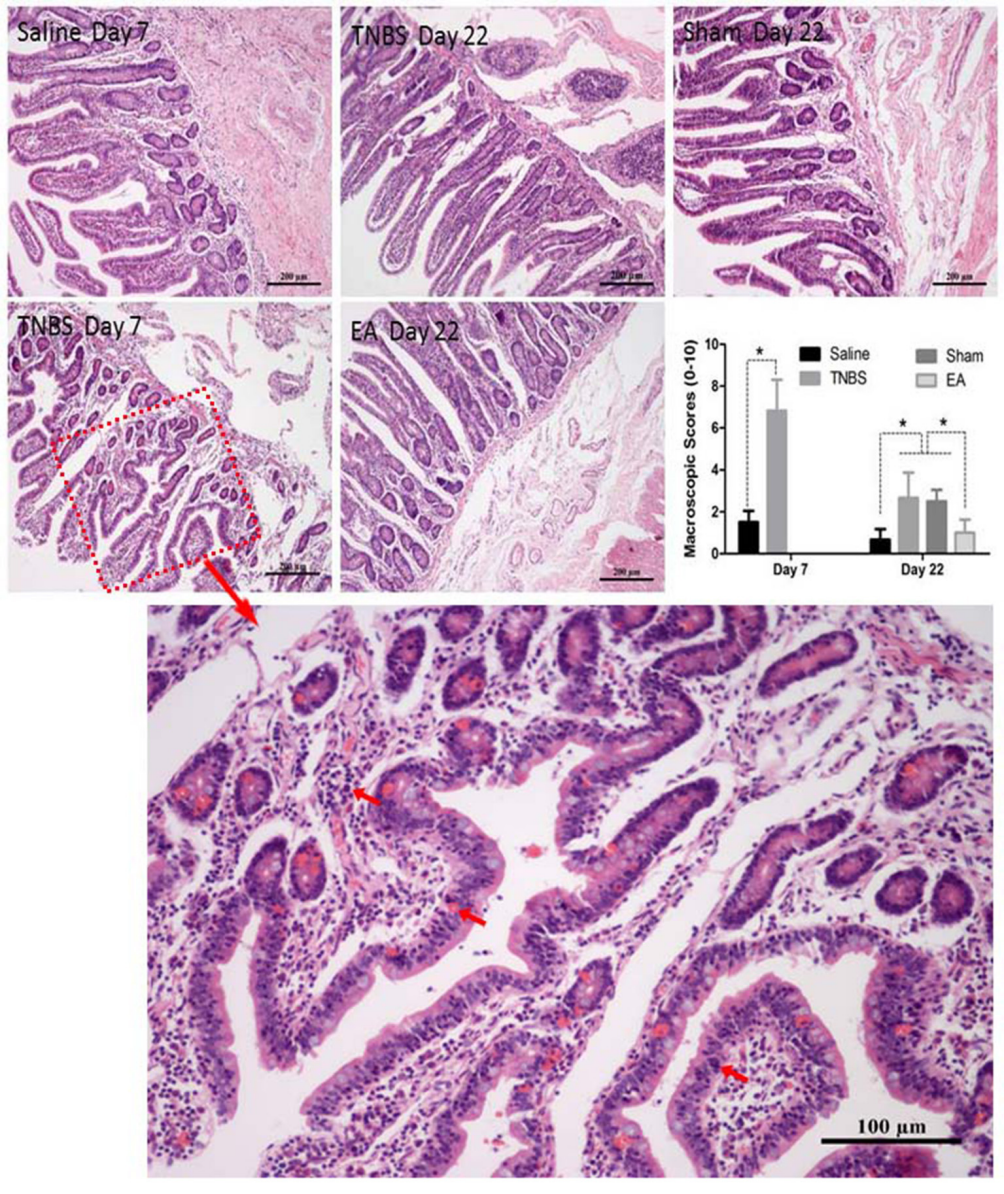
Effect of ileitis and EA treatments on microscopic pathologic changes. Microscopic changes of the ileum stained with hematoxylin and eosin (HE) at day 7 and 22. Arrows point to the inflammation cells or blood vessel congestion. The bars = 200 μm or 100 μm. The microscopic change scores on a scale of 0–10 at day 7 (mean ± SD, *n* = 3) and 22 (mean ± SD, *n* = 6), crypt depth (0–2), inflammatory cells (0–3), blood vessel congestion (0–3), ulceration (0–2). ^*^P < 0.05 Kruskal-Wallis analysis followed by Mann-Whitney *U*-test.

The saline-treated ileums showed no any apparent histopathological changes at day 7 and 22. However, TNBS-treated ileums revealed light red color, sporadic adhered pseudo-membrane, localized necrosis and apparent wall thickening at day 7. No apparent lesions were observed in the adjacent visceral organs and tissues such as large intestines, jejunum and mesentery (Figure 4B). Macroscopic lesions in TNBS group were more severe (*P* < 0.05) than those in saline group at day 7. However, no difference in macroscopic lesions among all groups was observed at day 22 (Figure 4C). Microscopically, TNBS-treated ileal wall showed extensive infiltration of neutrophils and lymphocytes in lamina propria, apparent submucosal and muscular layer ulceration and blood vessel congestion at day 7, and moderate inflammatory cell infiltration and granuloma in the submucosa and muscular layer at day 22. TNBS-treated goats showed severe (*P* < 0.05) microscopic lesions as compared with saline-treated goats at day 7 and 22. Sham or TNBS-treated goats showed severe (*P* < 0.05) microscopic lesions as compared with EA-treated goats at day 22. No difference in microscopic lesions between saline and EA group was observed at day 22 (Figure 5). TNBS-treated goats showed elevated (*P* < 0.05) MPO concentration as compared with saline-treated goats at day 7. However, no difference in the MPO concentration was observed among all groups at day 22 (Figure 4D).

### Effect of repeated EA treatments on VMR to CRD

**Figure 6:**
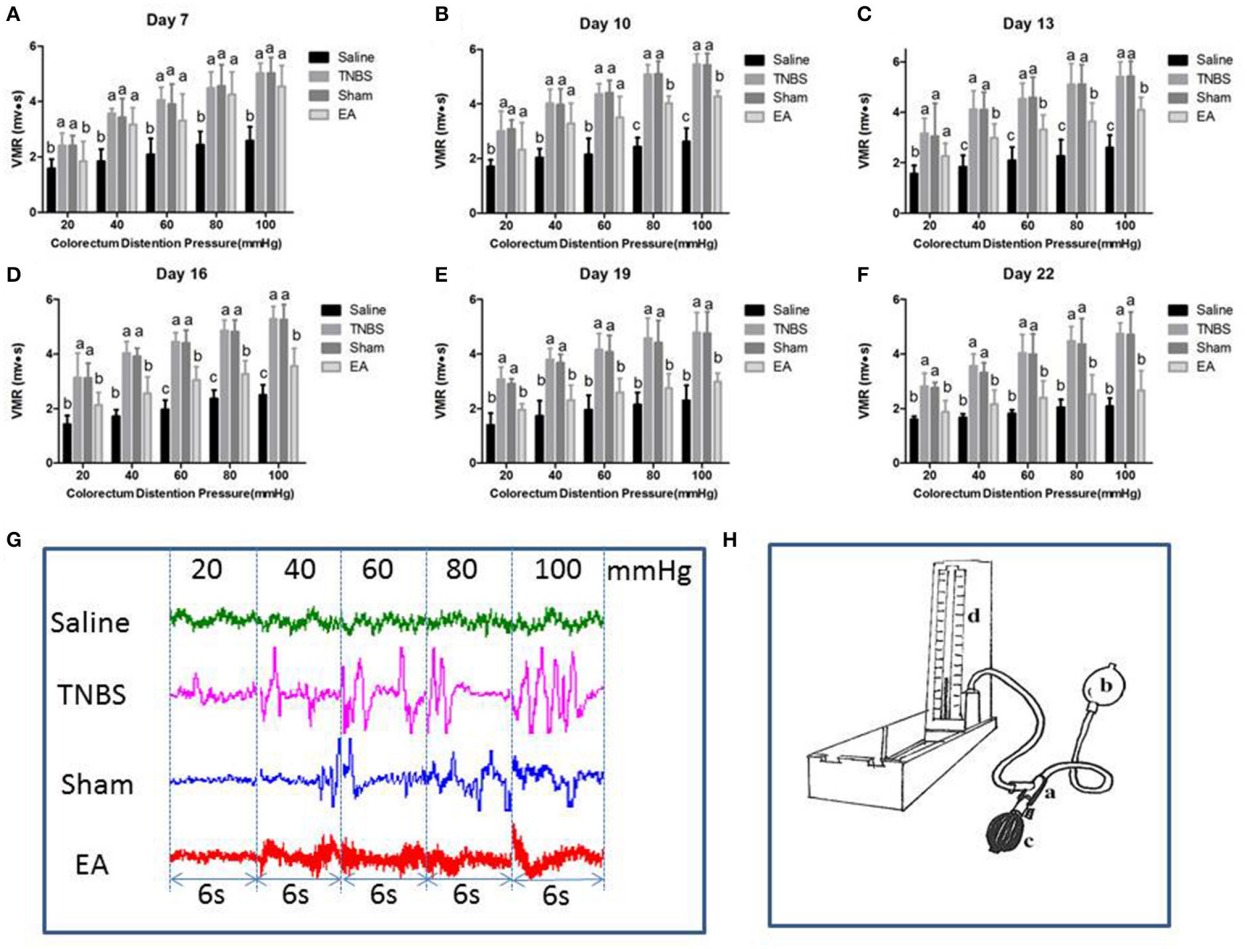
**(A–F)** Effects of repeated EA treatments on visceromotor responses (VMR) to colorectal distention pressure measured with electromyography (EMG) at day 7, 10, 13, 16, 19, and 22 (mean ± SD, *n* = 6). The values with different letters differ significantly (*P* < 0.05). One-way ANOVA followed by Bonferroni's post-test. **(G)** The representative EMG traces with 20, 40, 60, 80, and 100 mmHg distention pressures at day 16. The pressure continuously increased from 20 to 40, 60, 80, and 100 mmHg by stage and lasted for 6 seconds at each stage. **(H)** The distension device made with a T-connector (a), connecting a balloon (b), a vacuum pump (c) and a sphygmomanometer (d).

The EMG of abdominal muscles for assessment of VH is shown in Figure 6. The VMR was elevated with CRD stimulation intensity increasing. Compared to the goats in saline group, the goats in TNBS or sham group showed higher (*P* < 0.05) VMR to 20–100 mmHg distension pressures at day 7–22. Compared with saline-treated goats, EA-treated goats showed increased (*P* < 0.05) VMR to 40–100 mmHg distension pressures at day 7, to 20–100 mmHg distension pressures at day 10 and 13, and to 60–100 mmHg distension pressures at day 16. There was no difference in VMR between TNBS-treated and sham-treated goats during the experiment. Compared with sham or TNBS-treated goats, EA-treated goats showed decreased (*P* < 0.05) VMR to 20 mmHg distension pressure at day 7, to 80-100 mmHg distension pressures at day 10, to 40–100 mmHg distension pressures at day 13, and to 20–100 mmHg distension pressures at day 16–22.

### Effect of repeated EA treatment on pain behavioral response to CRD

**Figure 7:**
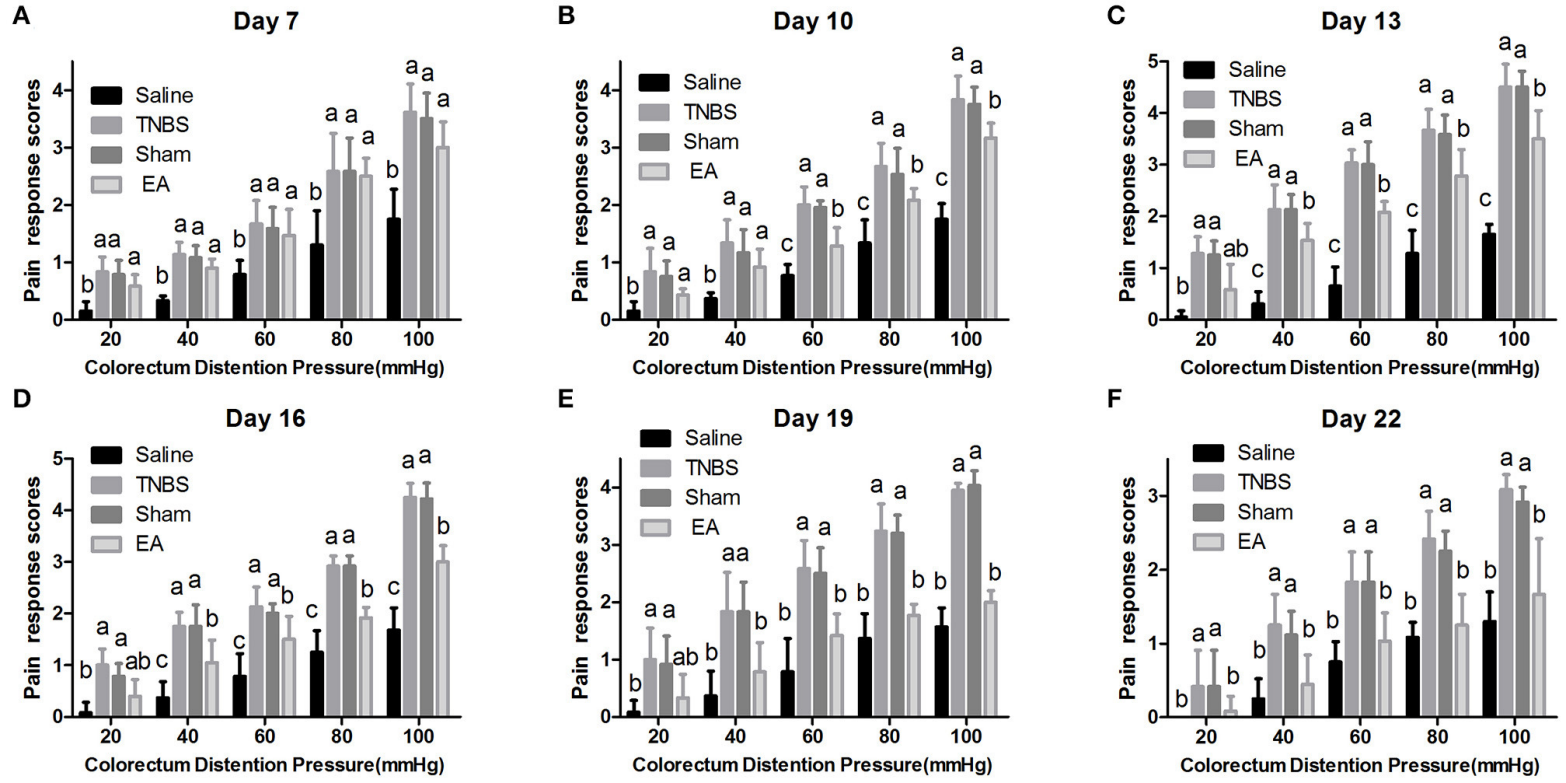
**(A–F)** Effects of repeated EA treatments on pain behavior response to colorectal distention pressure at day 7, 10, 13, 16, 19, and 22 (mean ± SD, *n* = 6). The pain response scores on a 0-4 scale, normal behavior-0, slightly modified behavior-1, mild behavior-2, moderate behavior-3, severe behavior-4. The values with different letters differ significantly (*P* < 0.05). Kruskal–Wallis analysis followed by Mann–Whitney *U*-test.

The experimental goats showed distinct signs such as restlessness, rapid breathing, guarding, tail wagging, curling of the lips, neck movement and change in posture when distension was applied. Compared with the saline-treated goats, Sham or TNBS-treated goats exhibited apparently increased (*P* < 0.05) behavioral responses to 20–100 mmHg distention pressures at day 7–22. EA-treated goats showed higher (*P* < 0.05) pain response scores than saline-treated goats to 20–100 mmHg distention pressures at day 7 and 10, and to 40–100 mmHg distention pressures at day 13 and 16. There was no difference (*P* > 0.05) in pain response scores to all distention pressures between TNBS and sham group during the experiment. Goats in EA group showed lower (*P* < 0.05) pain response scores than goats in TNBS group to 60–100 mmHg distention pressures at day 10, to 40–100 mmHg distention pressures at day 13–19, and to 20–100 mmHg distention pressures at day 22 (Figure 7).

### Effect of EA on the immunoreactivities of JAK2/STAT3 in the brain and spinal cord

**Figure 8:**
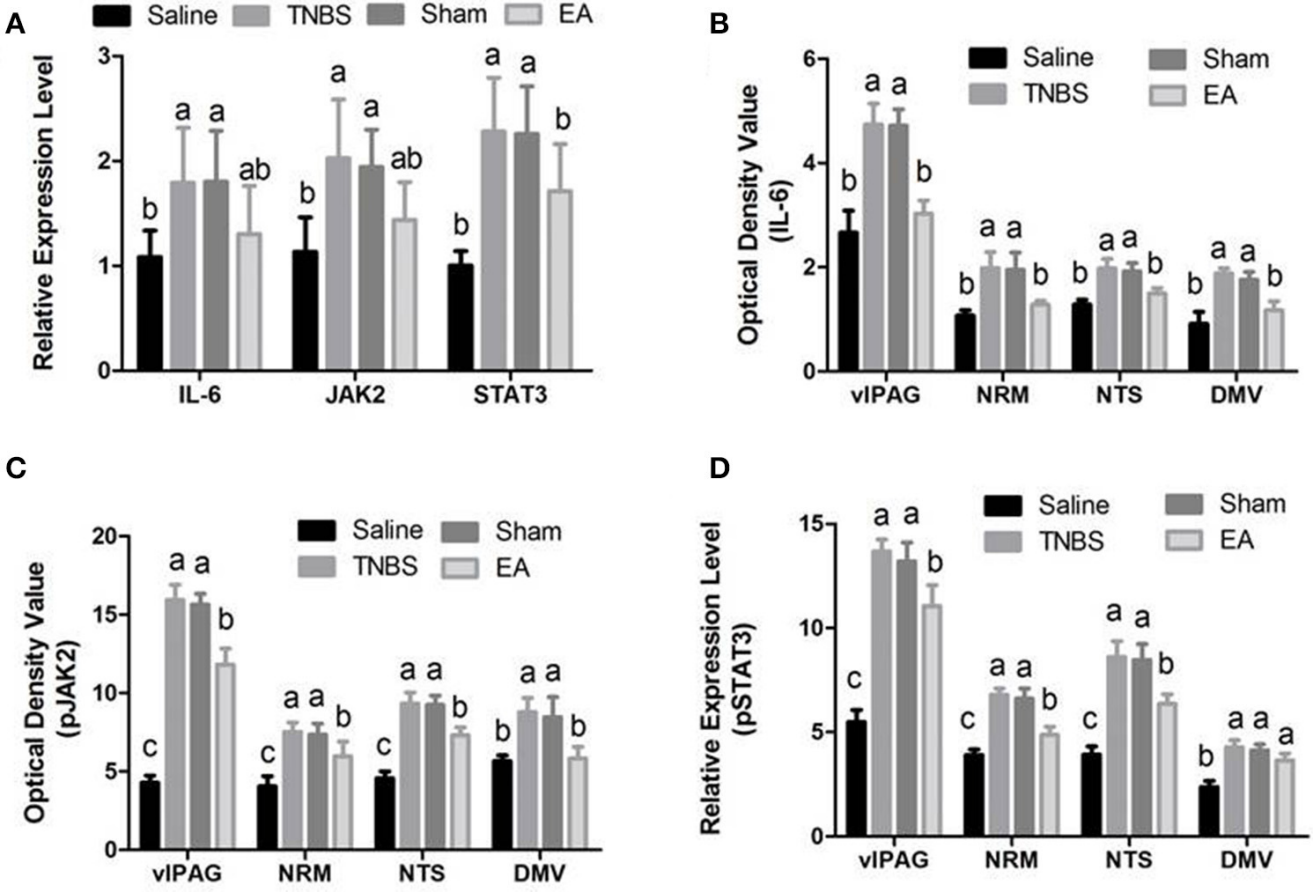
Effects of EA treatments on the expression of IL-6, JAK2, and STAT3 in the SCDH, vlPAG, NRM, NTS and DMV (mean ± SD, *n* = 6). **(A)** The mRNA expression of IL-6, JAK2 and STAT3 in the eleventh thoracic vertebra spinal cord dorsal horn (SCDH). **(B)** The protein expression of IL-6 in the ventrolateral periaqueductal gray (vlPAG), the nucleus raphe magnus (NRM), the nucleus tractus solitarius (NTS) and the dorsal motor nucleus of vagi (DMV). **(C)** The protein expression of phosphorylated JAK2 (pJAK2) in the vlPAG, NRM, NTS and DMV. **(D)** The protein expression of phosphorylated STAT3 (pSTAT3) in the vlPAG, NRM, NTS and DMV. The values with different letters differ significantly (*P* < 0.05). One-way ANOVA followed by Bonferroni's post-test.

The changes in IL-6, pJAK2 and pSTAT3 immunoreactivity-like cells were observed in vlPAG, NRM, NTS, DMV and SCDH. Their representative distributions are displayed in Figure 3. The expressions of IL-6, pJAK2 and pSTAT3 in the different treatments are shown in Figures 8B–D. Compared with saline-treated goats, TNBS-treated goats showed an increased (*P* < 0.05) expression in IL-6, pJAK2 and pSTAT3. Compared with the goats in saline group, the goats in EA group showed enhanced pJAK2 and pSTAT3 in vlPAG, NRM and NTS. There was no difference in the expression of these three substances between sham-treated and TNBS-treated goats. Compared with sham or TNBS-treated goats, EA-treated goats showed decreased (*P* < 0.05) IL-6, pJAK2 and pSTAT3 in the measured nuclei or areas except that in DMV where no change in pSTAT3 was observed.

### Effect of EA on JAK2/STAT3 expression in the spinal cord

Compared with saline-treated goats, TNBS-treated goats showed increased (P <0.05) gene expression of IL-6, JAK2 and STAT3. There was no difference in the gene expression of IL-6, JAK2 and STAT3 among sham-treated, TNBS-treated and EA-treated goats (Figure 8A).

**Figure 9:**
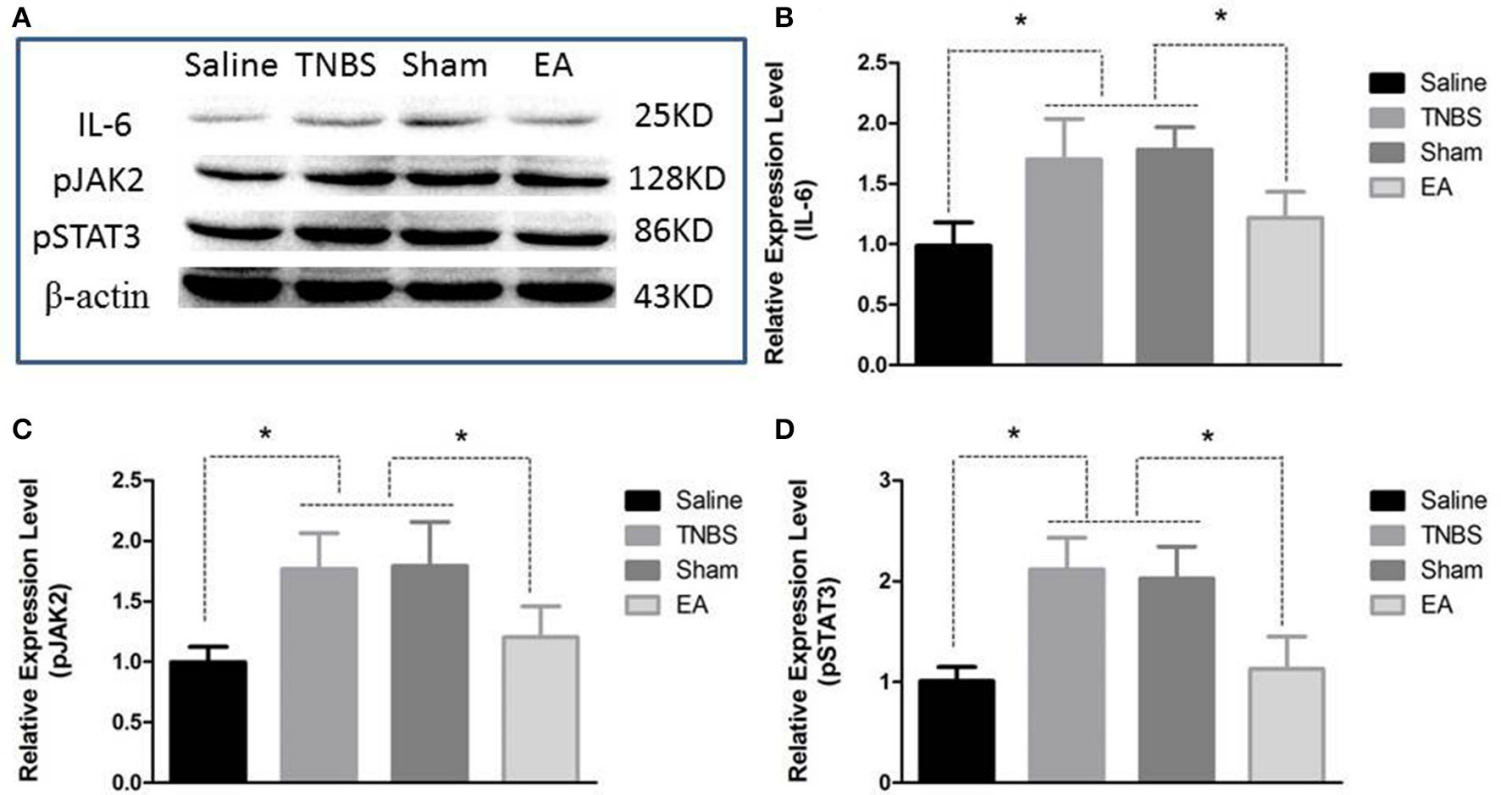
Effects of EA treatments on the protein expression of IL-6, pJAK2, and pSTAT3 in the eleventh thoracic vertebra spinal cord dorsal horn (mean ± SD, *n* = 6). **(A)** The western blotting bands. **(B)** IL-6 expression. **(C)** phosphorylated JAK2 (pJAK2) expression. **(D)** phosphorylated STAT3 (pSTAT3) expression. ^*^P < 0.05. One-way ANOVA followed by Bonferroni's post-test.

The protein level of IL-6, pJAK2 and pSTAT3 in the spinal cord are shown in Figure 9. Compared with saline-treated goats, TNBS-treated goats showed increased IL-6, pJAK2 and pSTAT3. There was no difference in the three proteins between saline-treated and EA-treated goats. IL-6, pJAK2 and pSTAT3 of EA goats were decreased compared with those of TNBS or Sham goats. There was no difference in the three proteins between TNBS and Sham goats.

## Discussion

VH is an important characteristic of IBD in human and animals. Although long-lasting pain states and chronic inflammation are reported to contribute to VH generation, its mechanism is not fully understood. Different models are utilized to study VH. Bradesi et al. (2005) placed rats in the condition of chronic water avoidance stress and established VH. Al–Chaer et al. (2000) gave colorectal distention to neonatal rats and developed visceral motility disturbances in their adulthood. Shah et al. (2016a) administered TNBS into rats' ileal lumen and developed ileitis-derived VH. Habibullah et al. (2016) and Hassan et al. (2015) injected TNBS into the ileal wall of goats and provoked visceral pain and VH, respectively. In the present study, 6 goats (3 in saline group and 3 in TNBS group) were used to verify if ileitis was induced at day 7. Another 24 goats were used for observing VH and EA intervention. The goats being administered with 30 mg TNBS in 40% ethanol exhibited inappetence, diarrhea, weight loss, increased MPO, and histopathologically apparent changes including mucosal congestion, hemorrhage, inflammation cell infiltration, wall thickening, adhesion, necrosis and ulceration at day 7. These symptoms and pathological lesions are consistent with the findings reported by Habibullah et al. (2016) and Hassan et al. (2015). The VMR and pain behavior response to CRD showed the visceral sensitivity occurred at day 7, with the maximum at day 13, and persisted to day 22, which is similar to the reports by Hassan et al. (2015) and Shah et al. (2016a).

EA has been identified as an effective treatment for various pains. The analgesic effect of EA application in VH has been reported (Cui et al., 2005; Liu et al., 2009b). The effect of EA depends on parameters such as its frequency, acupoints and interval. Cheng et al. (2012, 2013) demonstrated that 60 Hz EA induced the optimal analgesia effect in goats. In traditional Chinese medicine, acupoint at Zusanli is one of the most effective points for analgesia and gastrointestinal disorders (Han, 2003; Xu et al., 2009). Liu et al. (2009b) reported that EA at bilateral Zusanli for seven times significantly suppressed chronic VH. Wang et al. (2002) compared analgesic effects of EA treatments with different intervals (0, 1, 2 and 3 days) and found that EA with a 2-day interval exhibited the best cumulative analgesia effect. In the present study, 60 Hz EA with the 2-day interval at bilateral Housanli apparently alleviated ileitis-induced VMR and pain behavior response after its two interventions, and exhibited the maximal VH-relieving effect after its five treatments and the higher level at the termination of the experiment, which shows cumulative effect of EA on VH. These results are consistent with others' reports (Cui et al., 2005; Hassan et al., 2015; Shah et al., 2016a).

Acupuncture analgesia is essentially a manifestation of integrative processes at different levels in the CNS between afferent impulses from pain regions and impulses from acupoints. EA is verified to relieve pain through suppression (or activation) of the descending pain facilitatory (or inhibitatory) system mainly including PAG, RVM, and SCDH (Zhi-Qi, 2008). EA has been reported to regulate the vlPAG projecting to the RVM (mainly NRM) (Han et al., 1984; Xie et al., 2009; De et al., 2012; Coulombe et al., 2016), thereby to inhibit nociceptive impulse transmission of the SCDH to the higher center (Hurley et al., 2003; Li et al., 2007). Chu et al. (2012) and Zhuang et al. (2016) reported that peripheral and central inflammations induced an increase in proinflammatory cytokines (IL-6, IL-1β and TNF-α) and their receptor levels in the PAG and enhanced thresholds to mechanical and thermal stimuli. They further studied to find that the blockade or reduction of these cytokine receptors in the PAG restored the thresholds in rats. Liu et al. (2012) demonstrated that chronic pain provoked the up-regulation expression of proinflammatory cytokine (IL-6, IL-1β, and TNF-α) mRNAs in the RVM whereas the suppression of the cytokine production in the RVM reversed increased mechanical allodynia. Arruda et al. (2000) and Dominguez et al. (2008) found that chronic pain caused the increased concentration of IL-6 in SCDH and the antagonism to IL-6 with its specific antibody attenuated mechanical allodynia. These studies indicate the activation of these proinflammatory cytokines, especially IL-6 in the PAG-RVM-SCDH axis was involved in the development of pain hypersensitivity.

Recently, the molecular mechanism by which the CNS regulates chronic pain attracts more attentions. Numerous studies have confirmed that chronic pain evokes the activation of JAK2/STAT3 signaling pathway in the spinal dorsal horn of rats and thereby leads to an enhanced mechanical allodynia (Tsuda et al., 2011; Tang et al., 2012; Li et al., 2016; Liu et al., 2016). However, the blockade of JAK2/STAT3 signaling pathway with specific inhibitor attenuates the mechanical allodynia (Tsuda et al., 2011; Liu et al., 2016). Xue et al. (2014) found that neuropathic pain rats showed hypersensitivity along with the increased STAT3 mRNA and pSTAT3 but little change in the expression of STAT3. The specific inhibitor (WP1066) was shown to significantly inhibit either the STAT3 mRNA or the pSTAT3, but has no effect on the STAT3 protein level. These studies indicate that chronic pain induces hypersensitivity through activated JAK2/STAT3 (pJAK2 and pSTAT3), not JAK2 and STAT3 in the CNS. Dominguez et al. (2008) demonstrated the accumulation of neuropathic pain-induced pSTAT3 in the spinal dorsal horn coincided with an early and massive production of IL-6 mRNA in the DRG and an increase in IL-6 in the spinal dorsal horn, and immunoneutralization of IL-6 in the spinal dorsal horn led to a marked reduction of pSTAT3. Dominguez et al. (2010) and Wang et al. (2016) found that intrathecal administration of IL-6 rapidly activated JAK/STAT3 signaling pathway in the spinal dorsal horn and elevated mechanical allodynia in rats. Bradesi et al. (2015) reported that the increased expression of IL-6 transductor (gp130) and pSTAT3 in the spinal cord of rats was linked to chronic stress-induced VH. Wang et al. (2014) and Dominguez et al. (2010) demonstrated that the blockade of JAK2/STAT3 signaling pathway with the JAK2 inhibitor AG490, the STAT3 inhibitor S3I-201 or the suppressor of cytokine signaling (SOCS3) down-regulated the expression of IL-6 and relieved the pain hypersensitivity of rats. These studies indicate that IL-6 initiates the phosphorylation of JAK2 and STAT3, and in return the activation of the JAK2/STAT3 pathway promotes the production of IL-6. This vicious cycle is believed to lead to sustained central sensitization in response to chronic somatic and visceral nociception (Dominguez et al., 2008; Bradesi et al., 2015).

JAK2/STAT3 signaling pathway has been reported to involve the regulation of EA. Liu et al. (2012) found that EA inhibited the abnormal activation of JAK2/STAT3 signaling pathway in the cortex of rats with focal cerebral ischemia. Yan et al. (2015) reported that EA up-regulated the expression of pJAK2 and pSTAT3 in the liver tissue of obese rats. However, whether EA attenuates VH through JAK2/STAT3 signaling pathway in the pain descending system needs to be investigated. In the present study, a chronic pain and VH, and marked expression of IL-6, pJAK2 and pSTAT3 in the PAG-RVM-SCDH axis were induced by ileitis, but attenuated by the repeated EA stimulations. Our results did not show a change in the gene expression of IL-6, JAK2 and STAT3 after repeated EA treatments. This discrepancy between gene and protein expressions of IL-6, JAK2 and STAT3 may be caused by the inconsistent time-course of gene and protein expressions or post-transcription regulation. The post-transcription regulation of EA has been confirmed by Cui et al. (2017); EA induces miRNAs to modify the synthesis of targeted proteins. From our findings and others' reports, it is inferred that EA attenuates VH through modifying the activation of JAK2/STAT3 signaling pathway in the PAG-RVM-SCDH axis. Apart from JAK2/STAT3 signaling pathway, MAPKs (p38, ERK, JNK) (Galan et al., 2003; Kondo et al., 2013; Zhang et al., 2015) and PI3K/AKT (Kay et al., 2013; Bradesi et al., 2015) pathways were demonstrated to be activated in the VH. It warrants further investigation to understand whether EA alleviates VH via regulating these pathways or not.

VH is actually evoked by peripheral and central sensitization. Our study mainly focused on the modification of EA on the central sensitization although the model involved both intestinal hypersensitivity and inflammatory process. Wu et al. (2010) reported that EA treatment attenuated VH. Wu et al. (2007) reported that EA are effective for treating enteritis. However, whether EA suppressed ileal inflammation through regulating JAK2/STAT3 pathway or some other pathway is still need to be identified.

Abnormal gastrointestinal sensation and motility is commonly companied by VH in patients and animals. The NTS and the adjacent DMV, composing the dorsal vagal complex (DVC), is a primary modulating center for gastrointestinal motility. Nociception from the gastro-intestine transmits to the DVC, and then ascends to other upper areas such as PAG, to regulate gut function. Chen et al. (2013) reported that EA stimulation at Zusanli improved gastric motility in diabetic rats. Yang et al. (2014) found that EA at Zusanli had a promoting or inhibiting effect on the gastric motility through different signaling pathways. Wang et al. (2013) demonstrated that EA at Zusanli activated both excitatory and inhibitory gastric-related neurons in the NTS. In the present study, EA at Housanli relieved diarrhea, which is similar to other reports (Wang et al., 2013; Yang et al., 2014). Our study also showed that EA resulted in the inactivation of JAK2/STAT3 pathway in the NTS and DMV. However, whether EA relieves VH through regulating this pathway in the NTS or DMV is worthy to be investigated in the future.

## Conclusion

Repeated EA at bilateral Housanli effectively alleviated chronic VH probably through decreasing the expression level of IL-6, pJAK2 and pSTAT3 in the descending pain modulation system. EA could be a potential therapy against chronic VH.

## Author contributions

MD contributed to conception and design of the study. JW, YD, AT, MS, HJ, and XL performed animal experiments, collected samples and accomplished the laboratory investigations. YD, JW, and XL performed acquisition of data. YD, JW, and XL conducted data analysis and interpretation of data. JW drafted the manuscript. MD, YD, JZ, VV, and JW revised the manuscript. All authors read and approved the final manuscript.

### Conflict of interest statement

The authors declare that the research was conducted in the absence of any commercial or financial relationships that could be construed as a potential conflict of interest.
